# Estrogen induces c-Kit and an aggressive phenotype in a model of invasive lobular breast cancer

**DOI:** 10.1038/s41389-017-0002-x

**Published:** 2017-11-27

**Authors:** J. Chuck Harrell, Thomas M. Shroka, Britta M. Jacobsen

**Affiliations:** 1 Department of Pathology, Virginia Commonwealth University, Richmond, VA USA; 2 Department of Pathology, University of Colorado School of Medicine, Aurora, CO 80045 USA; 3 Department of Medicine, Division of Endocrinology, Metabolism and Diabetes, University of Colorado School of Medicine, Aurora, CO 80045 USA

## Abstract

Among the molecular subtypes of breast cancer are luminal (A or B) estrogen receptor positive (ER+), HER2+, and triple negative (basal-like). In addition to the molecular subtypes, there are 18 histologic breast cancer subtypes classified on appearance, including invasive lobular breast carcinoma (ILC), which are 8–15% of all breast cancers and are largely ER+ tumors. We used a new model of ER+ ILC, called BCK4. To determine the estrogen regulated genes in our ILC model, we examined BCK4 xenograft tumors from mice supplemented with or without estrogen using gene expression arrays. Approximately 3000 genes were regulated by estrogen in vivo. Hierarchical cluster analyses of the BCK4 derived tumors compared with ER+ and ER- breast cancer cell lines show the estrogen treated BCK4 tumors group with ER- breast cancers most likely due to a high proliferation score, while tumors from cellulose supplemented mice were more related to ER+ breast tumor cells. To elucidate genes regulated in vitro by estrogen in BCK4 cells, we performed expression profiling using Illumina arrays of the BCK4 cell line, treated with or without estrogen in vitro. A set of ~200 overlapping genes were regulated by estrogen in the BCK4 cell line and xenograft tumors, and pathway analysis revealed that the c-Kit pathway might be a target to reduce estrogen-induced proliferation. Subsequent studies found that inhibition of c-Kit activity using imatinib mesylate (Gleevec®) blocked estrogen mediated stimulation of BCK4 tumors and BCK4 cells in vitro as effectively as the anti-estrogen fulvestrant (Faslodex®). Decreased expression of c-Kit using shRNA also decreased baseline and estrogen induced proliferation in vitro and in vivo. These studies are the first to indicate that c-Kit inhibition is an effective approach to target c-Kit+ ILC.

## Introduction

There are at least 18 different histological subtypes of breast cancer. Among these are invasive breast carcinoma of no special type (IC-NST, formerly known as invasive ductal carcinoma), invasive lobular carcinoma (ILC) which comprise 8–15% of all breast tumors, and mucin-secreting mucinous breast cancers (MBC; >90% mucin) that comprise ~4% of all breast cancers. Most MBC are considered ductal in origin because of their secretion of extracellular mucin, however, there are several recent reports of ILC that produce extracellular mucus^1–3^, and expression profiling of 11 histological subtypes shows some mucinous tumors are similar to ILC^4^, suggesting these breast cancer subtypes may be related. In fact, ILC can be further stratified into subtypes including classic and non-classic (including pleomorphic and mucinous subtypes, reviewed in ref. ^5^). Histological stratification of ILC is important as patients with non-classic ILC have worse overall survival and disease-free survival compared to classic ILC^6^. The presence of signet ring (SR) cells (so named because of displacement of the nucleus from the intracytoplasmic containment of mucin) may or may not be noted by pathologists if the cells account for less than 20% of the tumor volume. The prevalence of SR cells may be clinically relevant because patients with ILC tumors containing >10% signet ring cells typically have more aggressive tumors with a worse overall survival than patients that lack SR cells^7^. Importantly, pleomorphic ILC (pILC) commonly contain SR cells^5^. While breast tumors containing signet ring cells are commonly lobular^8^, other histological types of breast cancer (IC-NST or MBC) may also contain signet ring cells^9^ and patients with tumors containing SR cells have a higher incidence and number of lymph node metastasis and higher mortality than patients with tumors lacking SR cells^9^.

ILC are typically ER+ (>90%) and/or PR+ (70–80%) but usually lack HER2 overexpression^10, 11^. ILC also tend to be diploid with low proliferative index^10^, however, ILC tend to spread in a diffuse pattern making it difficult to resect the tumor margins (reviewed in ref. ^12^). Metastases in patients with ILC often manifest in bone and lung as they do with IC-NST, however, ILC also metastasize to the abdominal cavity (reviewed in ref. ^12^). Models to study ER+ ILC are rare; to date there are only 3 models, the MDA-MB-134VI and SUM44PE cells and our recently developed BCK4 cells^13^, that form ILC with mucinous features upon supplementation with estrogen. BCK4 cells are designated as lobular based on their lack of e-cadherin and cytoplasmic localization of p120 (delta catenin)^13^, they contain SR cells and are GCDFP-15 positive indicating they may represent the pleomorphic subtype of ILC^14^.

One protein expressed in many ILC and pILC is c-Kit/CD117^15, 16^. C-Kit is a receptor tyrosine kinase activated by its cognate ligand, kit ligand (KITL), and is involved in regulation of hematopoiesis. Mutations in c-Kit that increase the binding of the c-Kit inhibitor, imatinib mesylate, commonly occur in gastrointestinal stromal tumors. Within the mammary gland c-Kit is expressed during mammary gland development in normal epithelial cells both within the duct and terminal ductal lobular units^17^, then decreases in invasive breast tumors^18^. However, expression of c-Kit in breast tumors in general is controversial. Among over 1600 breast tumors examined for c-Kit with IHC, only 2.6% of breast tumors were positive for c-Kit^19^. Another cohort examining 924 breast tumors showed 14.7% contained c-Kit^20^ where its expression correlated with a higher incidence of metastasis and poor patient outcome. Among 112 breast tumors of histological special types, c-Kit was not detected in MBC nor ILC^4^ while in a larger study of 1600 breast tumors examined on breast tumor microarrays, c-Kit was detected in 2% of MBC and 0.5% of ILC^19^. However, specifically among pILC, 15.4% of tumors contained c-Kit^16^, and a recent study examining 147 ILC tumors by RPPA analysis shows ~30% of all ILC express c-Kit^15^. With regard to ER status, there are several reports of c-Kit expression in ER- breast tumors^4, 21^ where c-Kit expression correlates with a poor prognosis^21^. Expression of c-Kit is also detected among ER+ breast tumors, where its expression ranges from 14% to 40%^22, 23^; one study reports c-Kit expression is higher in ER+ than ER- breast tumors^24^. Estrogen mediated regulation of c-Kit has not been examined in the mammary gland nor breast cancer, although c-Kit is hormonally regulated in the normal ovary (reviewed in ref. ^25^).

Numerous studies have profiled estradiol (E2) regulated genes in IC-NST (reviewed in ref. ^26^) and one study of ILC^27^. However, what genes are E2 regulated in a model of pILC or in a model of ILC in vivo has not been reported. In these studies we examined E2 mediated gene regulation in our pILC models both in vitro and in vivo. While many of the genes overlap between cells and tumors, some are unique. We wished to compare E2 regulated genes in our ILC model to those previously reported in the other two ER+ ILC cell lines. Furthermore, while some of the genes overlap with those regulated in other ER+ breast cancer cell lines, many genes are unique to pILC cells. Elucidation of genes regulated by E2 in pILC may provide novel targets to treat patients with lobular breast tumors. We show a unique subset of genes are regulated by E2 in BCK4 cells, among these is c-Kit.

## Results

### Intrinsic subtype analysis of BCK4 cells

**Fig. 1:**
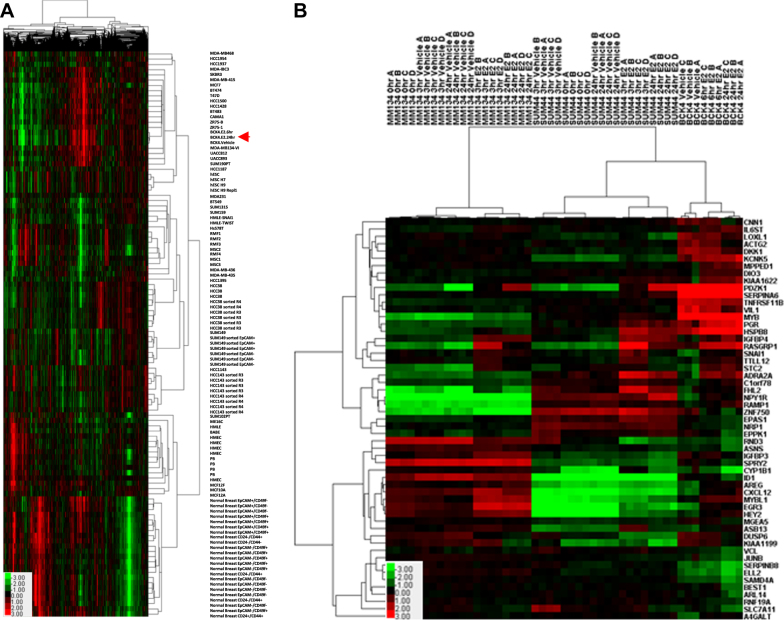
**Gene regulation in BCK4 cells. a** Supervised clustering of BCK4 cells vs. other breast cancer cell lines using 4000 intrinsic genes^28^. **b** Expression profiling data of BCK4 cells treated without or with estsradiol (E2) for 6 or 24 h; the most significant 55 estrogen regulated genes are shown in comparison with SUM44PE (SUM44) and MDA-MB-134VI (MM134) cells treated with vehicle or estradiol (E2) for 3 or 24 h. Heat maps show genes with high (red), unchanged (black) and low (green) expressions

BCK4 cells have been in culture a relatively short time compared with other widely used breast cancer cell lines, the majority of which were established in the 1970s. We compared the BCK4 cells treated with vehicle or E2 for 24 h with a panel of breast cancer cell lines and normal breast samples using 4000 intrinsic genes^28^ to examine relatedness among the samples. As shown in Fig. 1a, BCK4 cells cluster with other ER+ breast cancer cell lines with a positive node correlation (>0.5). Importantly the BCK4 cells cluster more closely with each other (node correlation >0.96) compared to any other breast cancer cell lines. We next assessed the relative differentiation status of these cell lines^29, 30^. Interestingly, the Differentiation Score of the BCK4 cells was significantly higher than all other ER+ breast cancer cell lines, including the “normal” breast epithelial cell lines MCF10A and MCF12A (Table 1). Treatment of BCK4 cells with 24 h of E2 increased their proliferation rate (Table 1), while not affecting their Differentiation Score. This shows that BCK4 cells have similarities with normal cells and slower growing luminal cancers, reflecting the more differentiated state of BCK4 cells than other ER+ breast cancer cell lines.

**Table 1:** Assessment of differentiation and proliferation scores across human breast cancer cell lines

Gene expression microarray	Differentiation score	Proliferation score
Normal Breast Sort 3 EpCAM+/CD49f- R8	0.47	−3.76
Normal Breast Sort 5 EpCAM+/CD49f- R6	0.43	−4.03
Normal Breast Sort 4 EpCAM+/CD49f- R8	0.38	−3.51
BCK4.EtOH.6hr2	0.36	0.12
BCK4.E2.24hr2	0.35	0.26
BCK4.E2.6hr2	0.33	0.11
BT474 cell line	0.31	0.15
ZR75-1 cell line	0.30	0.12
SUM190PT cell line	0.30	0.02
MDA-MB-415	0.29	0.17
MCF7 cell line	0.29	−1.34
T47D p8	0.28	0.65
UACC893 p47	0.28	−0.38
HCC1500 cell line p2	0.27	0.37
UACC812 cell line	0.26	−0.38
MDA−IBC3 cell line	0.24	0.19
BT483 cell line	0.23	−0.32
HCC1428 cell line p12	0.22	0.80
ZR75-B	0.21	0.73
SKBR3 cell line p17	0.21	0.61
MDA-MB134-VI	0.21	−0.31
Normal Breast Sort CD24+CD44+ 2/13/09	0.21	−2.73
CAMA1 cell line	0.21	0.56
HCC1954 cell_line	0.20	−0.81
MDA-MB468 parental cell line p13	0.11	0.87
HCC1937 cell line	0.08	1.04
MCF12F	0.08	−1.53
Normal Breast Sort 3 EpCAM+/CD49f+ R9	0.07	−2.95
HCC1143 sorted R3 Exp2	0.06	1.25
Normal Breast Sort EpCAM+/CD49f+ P5	0.05	−2.17
HCC1143 sorted R3 Exp6	0.05	0.77
Normal Breast Sort 2 EpCAM+/CD49f+ P5	0.05	−2.30
HCC38 sorted R3 Exp2	0.04	0.45
HCC38 sorted R3 Exp3	0.04	0.80
SUM149 sorted 5b/R6 HuMEC CD49f+/EpCAM+ --Exp1	0.04	1.06
SUM149 sorted 5a/R1 HuMEC CD49f+/EpCAM+ --Exp1	0.04	1.13
Normal Breast Sort 5 EpCAM+/CD49f+ R8	0.04	−3.93
HCC38 sorted R3 Exp4	0.03	0.78
HCC1187 cell line	0.03	0.60
HCC1143 p11	0.03	0.38
MB231 cell line p2 DMEM	0.03	1.06
Normal Breast Sort 4 EpCAM+/CD49f+ R9	0.03	−3.31
SUM149 sorted 6a/R6 HuMEC+FBS CD49f+/EpCAM+ --Exp1	0.02	1.39
SUM149 p12 HuMEC+5%FBS	0.01	1.10
HCC1143 sorted R3 Exp3	−0.02	1.17
HCC1143 sorted R3 Exp5	−0.02	1.08
HCC38 sorted R3 Exp1	−0.02	1.48
MCF10A	−0.02	−0.02
HCC38 sorted R4 Exp3	−0.03	0.44
Mani-HMLE_HuMEC	−0.03	0.52
HCC38 sorted R4 Exp4	−0.03	0.39
HCC38 cell line	−0.03	0.39
SUM102PT cell line	−0.04	−2.64
SUM149 sorted 6a/R5 HuMEC+FBS CD49f+/EpCAM- --Exp1	−0.04	1.15
HCC1143 sorted R4 Exp6	−0.04	0.12
Normal Breast Sort 2 CD24-/CD44- R6-	−0.04	−3.15
HCC38 sorted R4 Exp2	−0.05	0.71
HCC1143 sorted R4 Exp2	−0.06	1.38
MDA-MB-435	−0.06	0.93
HCC38 sorted R4 Exp1	−0.06	1.04
SUM149 sorted 5b/R5 HuMEC CD49f+/EpCAM- --Exp1	−0.06	1.51
SUM149 sorted 5a/R6 HuMEC CD49f+/EpCAM- --Exp1	−0.06	1.58
BABE cell line	−0.07	−1.05
PB-p48	−0.08	−2.59
PB-p52	−0.08	−1.53
ME16C cell line	−0.08	−0.24
Mesench. Stem Cell 3	−0.09	−0.34
SUM1315 cell line	−0.09	0.40
MDA-MB436 cell line	−0.09	0.70
HCC1395 cell line	−0.10	0.56
PB-p78	−0.11	−2.87
HCC1143 sorted R4 Exp5	−0.11	0.74
HMEC CB	−0.11	0.46
RFM4-Kuperwasser	−0.11	−1.80
HMEC BW HuMEC p2	−0.12	−2.68
Mani-HMLE-TWIST1	−0.12	1.10
MCF12A	−0.12	0.82
hESC H9	−0.12	0.46
hESC H7 -- Repl1	−0.12	0.14
hESC H9 Repl1	−0.12	0.40
hESC H7	−0.12	0.14
BT549 cell line	−0.12	1.30
HCC1143 sorted R4 Exp3	−0.12	0.87
Hs578T p11	−0.14	−0.22
Normal Breast Sort 2 CD24-/CD44+ R7-	−0.14	−3.64
SUM159 p12	−0.16	1.11
Normal Breast Sort 3 EpCAM-/CD49f- R7	−0.16	−2.83
PB-p86	−0.16	0.53
Normal Breast Sort 4 EpCAM-/CD49f- R7	−0.16	−3.22
HMEC BL* p2	−0.16	−0.31
HMEC CA	−0.17	−0.69
Mesench. Stem Cell 2	−0.18	−2.08
Normal Breast Sort 5 EpCAM-/CD49f- R7	−0.19	−3.18
Normal Breast Sort 2 EpCAM-/CD49f- P4	−0.19	−3.28
RFM3 p4	−0.19	−1.02
Mani-HMLE-SNAI1	−0.19	1.35
RFM2 p4	−0.20	−0.90
Mesench. Stem Cell 1	−0.20	−0.01
RFM1 p4	−0.20	−0.60
HMEC BX HuMEC p2	−0.21	0.36
Normal Breast Sort EpCAM-/CD49f- P4	−0.21	−3.47
Normal Breast Sort 3 CD24-/CD44+	−0.27	−2.93
Normal Breast Sort 4 EpCAM-/CD49f+ R6	−0.29	−3.87
Normal Breast Sort CD24-CD44+ 2/13/09	−0.29	−3.33
Normal Breast Sort 3 EpCAM-/CD49f+ R6	−0.30	−2.67
Normal Breast Sort EpCAM-/CD49f+ P6	−0.31	−3.57
Normal Breast Sort 2 EpCAM-/CD49f+ P6	−0.33	−3.94
Normal Breast Sort 5 EpCAM-/CD49f+ R5	−0.37	−3.96

### Estrogen regulated genes in the BCK4 cell line

To determine the panel of E2 regulated genes in BCK4 cells, the cells were treated with or without estrogen (E2) for 6 or 24 h and expression profiling was performed. Figure 1b shows the 55 most significantly regulated genes when our dataset was combined with studies examining the E2 regulated genes identified by Sikora et al. in two ER+ ILC cell lines, MDA-MB-134VI and SUM44PE^31^. Among these are classically estrogen regulated genes, including progesterone receptor (PGR), IL6ST, CXCL12, MYBL1 and MYB. Estrogen also regulated some unique genes in BCK4 cells including A4GALT and DKK1. In BCK4 cells, more genes are regulated by E2 at the 24 than 6 h timepoint in agreement with Sikora et al. in the other ER+ ILC cells (see Supplementary Table 1 and ref. ^31^). All E2 regulated genes in BCK4 cells are listed in Supplementary Table 1. Supervised cluster analysis shows E2 mediated gene regulation is more similar between BCK4 and SUM44PE (SUM44) cells than BCK4 and MDA-MB-134VI (MM134) cells; however, many genes are regulated by E2 in both BCK4 and MM134 cells including MYBL1, IL6ST and EGR3 (Supplementary Table 1). Several genes are E2 regulated in all 3 ILC cell lines such as PDZK1, RASGRP1 and SNAI1. However, most of the genes commonly E2 regulated among all 3 ILC cell lines are not unique to ILC as many IC-NST cell lines also regulate these genes (Supplementary Table 1 and Sikora et al.^31^). Differences in the panel of genes regulated by E2 among the ILC cell lines likely reflects the molecular heterogeneity of ILC^6, 15^.

### Estrogen regulated genes in BCK4 tumors

**Fig. 2:**
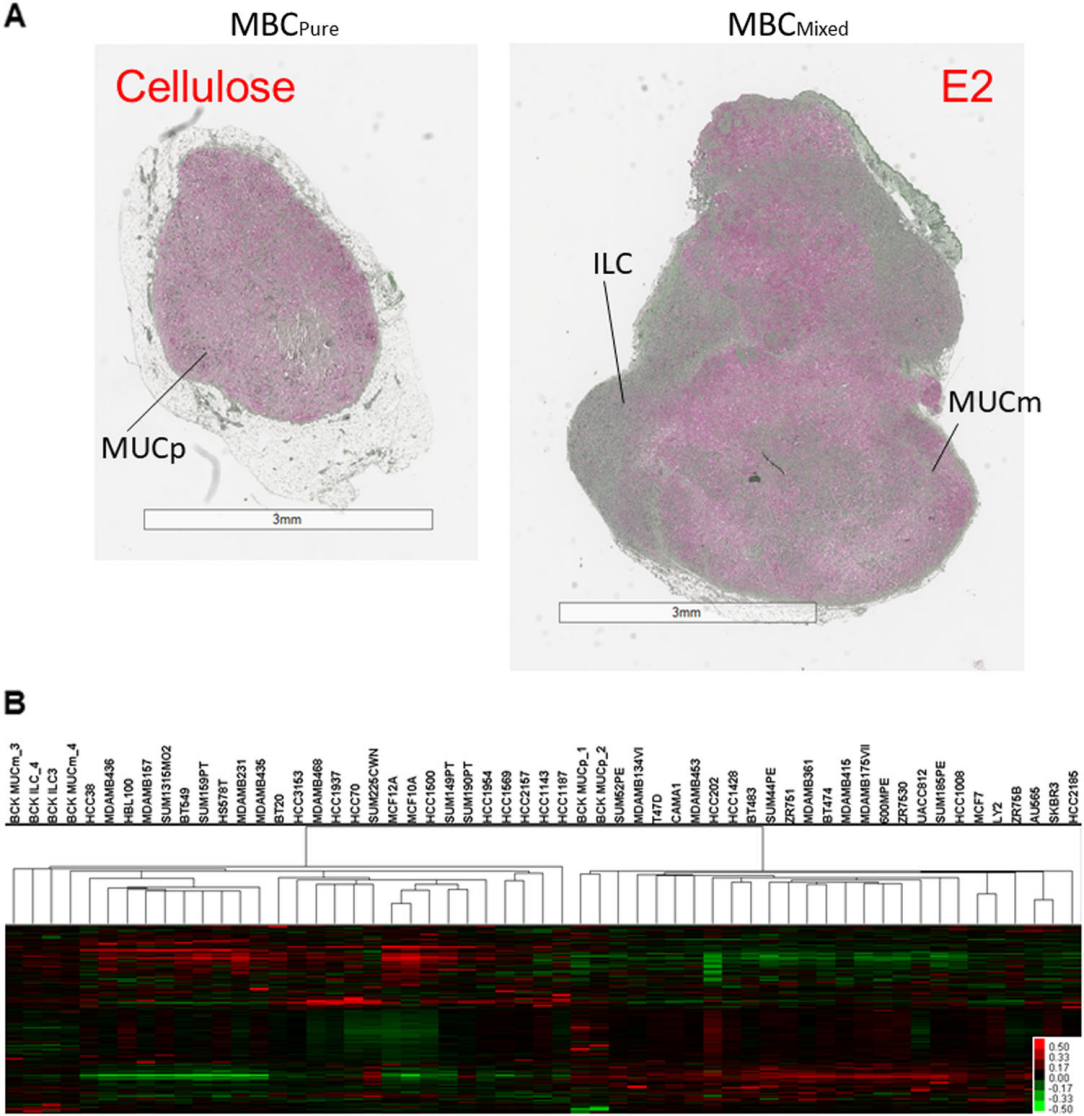
**a** BCK4 tumors stained with mucicarmine to examine genes regulated among tumor regions. Tumors were stained with mucicarmine (pink) and counterstained with apple green. Pure mucinous (MUCp) or mixed mucinous (MUCm) and ILC regions are shown. **b** Supervised hierarchical clustering of 11,358 overlapping genes in BCK4 tumor regions vs. breast cancer cell lines. Heat map shows genes with relatively high (red), unchanged (black), and low (green) expressions

Estrogen treatment induces a histologic change in tumors derived from BCK4 cells; in the absence of E2 supplementation, BCK4 cells form highly mucinous tumors (pure mucinous: MUCp). Upon addition of E2, tumors showed a mixed mucinous morphology with some mucinous tumor regions (MUCm) and some regions containing tightly packed tumor cells with less mucin (ILC) (Fig. 2a and Supplementary Fig. 1). To elucidate the mechanism(s) underlying this histologic switch, we performed gene expression profiling of the different tumor regions using LASER Capture Microdissection from the pure tumor (MUCp), and the two regions of the mixed tumor (MUCm and ILC regions).

The data were then combined with the gene expression data from Neve et al.^32^ to understand if these regions yielded distinct genetic profiles. Similar to the estrogen-induced proliferation of the BCK4 cells observed in vitro (Fig. 1b), the genetic analysis of the different tumor regions found that the ILC region of the BCK tumors were considerably more mitotically active than the mucin secreting cells, with proliferation scores similar to that found in Basal-like cells. However, the differentiation scores remained in the luminal range suggesting that the aggressive component of these tumors is luminal-like and estrogen sensitive (Table 2). Hierarchical cluster analysis showed that the pure mucinous tumors are more similar to the ER+ breast cancer cells, while the E2 treated tumor regions (either MUCm or ILC) cluster within the ER- breast cancer cells (despite the fact the tumors retain ER and PR^13^); this is likely driven by the high proliferation rates of the ILC region of BCK4 tumors (Fig. 2b).

**Table 2:** Assessment of differentiation and proliferation scores of different regions of BCK tumors compared with a second breast cancer cell line database

Array	Differentiation score	Proliferation score
600MPE	0.15	−0.62
AU565	0.14	−0.46
BCK.ILC_3	−0.03	0.51
BCK.ILC_4	−0.03	1.07
BCK.MUCp_1	0.03	−0.88
BCK.MUCp_2	0.02	−0.97
BCK.MUCm_3	−0.04	−0.02
BCK.MUCm_4	−0.05	−0.02
BT20	−0.08	0.16
BT474	0.15	−0.51
BT483	0.19	−0.74
BT549	−0.27	0.85
CAMA1	0.12	−0.63
HBL100	−0.18	0.74
HCC1008	0.15	−0.58
HCC1143	−0.10	−0.42
HCC1187	−0.12	0.28
HCC1428	0.06	−0.25
HCC1500	−0.20	0.16
HCC1569	−0.19	−0.08
HCC1937	−0.06	0.02
HCC1954	−0.08	−0.23
HCC202	0.14	−0.81
HCC2157	−0.15	−0.21
HCC2185	0.10	−0.55
HCC3153	−0.12	−0.32
HCC38	−0.13	−0.15
HCC70	−0.01	−0.03
HS578T	−0.26	0.64
LY2	−0.02	−0.11
MCF10A	−0.30	0.00
MCF12A	−0.20	0.04
MCF7	0.09	−0.22
MDAMB134VI	0.10	−0.15
MDAMB157	−0.22	0.57
MDAMB175VII	0.20	−0.55
MDAMB231	−0.22	1.12
MDAMB361	0.20	−0.62
MDAMB415	0.19	−0.10
MDAMB435	−0.21	0.46
MDAMB436	−0.18	0.54
MDAMB453	0.08	−0.36
MDAMB468	−0.04	0.09
SKBR3	0.07	−0.72
SUM1315MO2	−0.23	0.44
SUM149PT	−0.12	0.43
SUM159PT	−0.29	0.78
SUM185PE	0.10	−1.20
SUM190PT	0.06	−0.27
SUM225CWN	0.15	−0.51
SUM44PE	0.20	−0.92
SUM52PE	0.05	−0.87
T47D	0.08	−0.12
UACC812	0.21	−0.40
ZR751	0.17	−0.17
ZR7530	0.17	−0.53
ZR75B	0.07	−0.08

### Genes regulated in BCK4 derived tumors vs. BCK4 cell lines

We also analyzed E2 regulated genes common between BCK4 cells and BCK4-derived tumors. Using a SAM q value of ≤5 we identified 356 E2 regulated genes regulated in both BCK4 cells and tumors (Supplementary Fig. 2, Supplementary Table 2). Two hundred fifty three genes were unique in BCK4 cells showing no overlap with genes regulated in BCK4 xenografts (which may be a reflection of short E2 treatment timepoint of 24 h in the cells vs. ~5 months in the tumors). Among the BCK4 tumors, the largest number of differentially expressed genes was observed when comparing the ILC tumor region vs. MUCp. There was significant overlap among genes regulated in the different tumor regions (for gene lists see Supplementary Table 2).

**Fig. 3:**
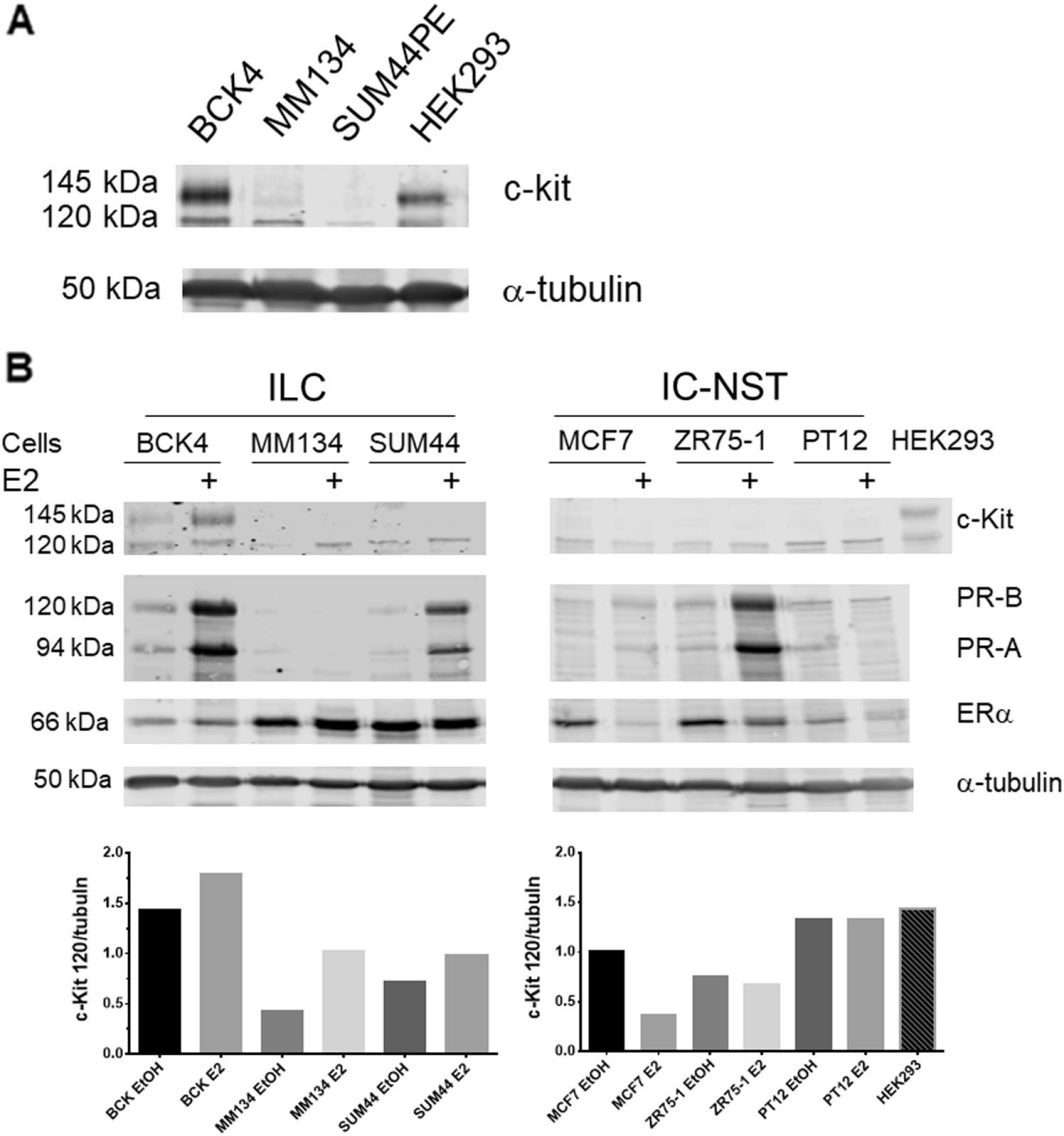
**a** Western blot of ILC cells grown under regular conditions showing baseline levels of c-Kit. Tubulin is shown as a loading control. **b** Western blot showing c-Kit expression in control or estradiol (E2) or vehicle (EtOH) treated ILC cells: BCK4, MDA-MB-134VI (MM134) and SUM44PE (SUM44) and IC-NST cells: MCF7, ZR75-1 and PT12. HEK-293 cells are shown as a control for c-Kit expression: the c-Kit antibody recognizes both the 120 and 140 KDa isoforms of c-Kit. Also shown is PR (PR-A and PR-B) induction mediated by E2 in ER+ breast cancer cell lines and ER expression.. Alpha tubulin is shown as a loading control. Densitometry shows quantification of the level of p120 c-Kit corrected to tubulin for ILC and IC-NST cell lines

Next we examined pathways regulated among the comparisons of the tumor regions (Supplementary Fig. 3). MetaCore pathway analysis shows the top 16 enriched pathways among the MUCp and ILC tumor regions. Among these is the signaling pathway for c-Kit. Among the regulated genes are not only c-Kit itself, but also the ligand for c-Kit (KITLG) and other components of the c-Kit signaling pathway including SHP2, PDK, and SOS. To confirm regulation of the c-Kit protein by estrogen, we treated BCK4 cells for 1, 2, 7 or 14 days with or without E2. C-Kit is induced by E2 as early as 1 day (Supplementary Fig. 4). We also examined regulation of ERα over the timecourse and observed ER does not downregulate with E2 in BCK4 cells. Connexin-43 (Cx43), another strongly E2 regulated gene in both BCK4 cells and tumors (Supplementary Table 1) also showed strong upregulation with E2 (Supplementary Fig. 4), confirming our microarray data. To examine regulation of c-Kit in other ER+ ILC and IC-NST cell lines, we treated cells with or without E2 for 7 days. Figure 3a shows the basal expression of c-kit in the ILC cell lines. Figure 3b shows E2 induction of both the 120 and 145 kDa forms of c-Kit in BCK4 cells. E2 also upregulated the 120 kDa isoform of c-Kit in MDA-MB-134VI and SUM44PE ILC cells. We also examined E2 regulation of c-Kit in IC-NST breast cancer cell lines MCF7, ZR75-1 and PT12; expression was not induced by E2 in any of the IC-NST lines. We also examined induction of the two PR isoforms, PR-A and PR-B, by E2 in the 3 ILC cell lines. PR is strongly induced by E2 in BCK4, SUM44PE, MCF7 and ZR75-1 cells, but not PT12 or MDA-MB-134VI (Fig. 3b), as previously reported^33^. Interestingly, ERα does not downregulate in any of the ILC cell lines upon treatment with E2, however, ERα is downregulated by E2 in all three IC-NST cell lines. We also confirmed regulation of c-Kit and Cx43 in BCK4 tumors; c-Kit is induced by E2 in both the mucinous and ILC region of the mixed tumor (Supplementary Fig. 5). Cx43 is strongly induced in the ILC region. In BCK4 tumors, ERα does not downregulate upon E2 treatment (Supplementary Fig. 5).

**Fig. 4:**
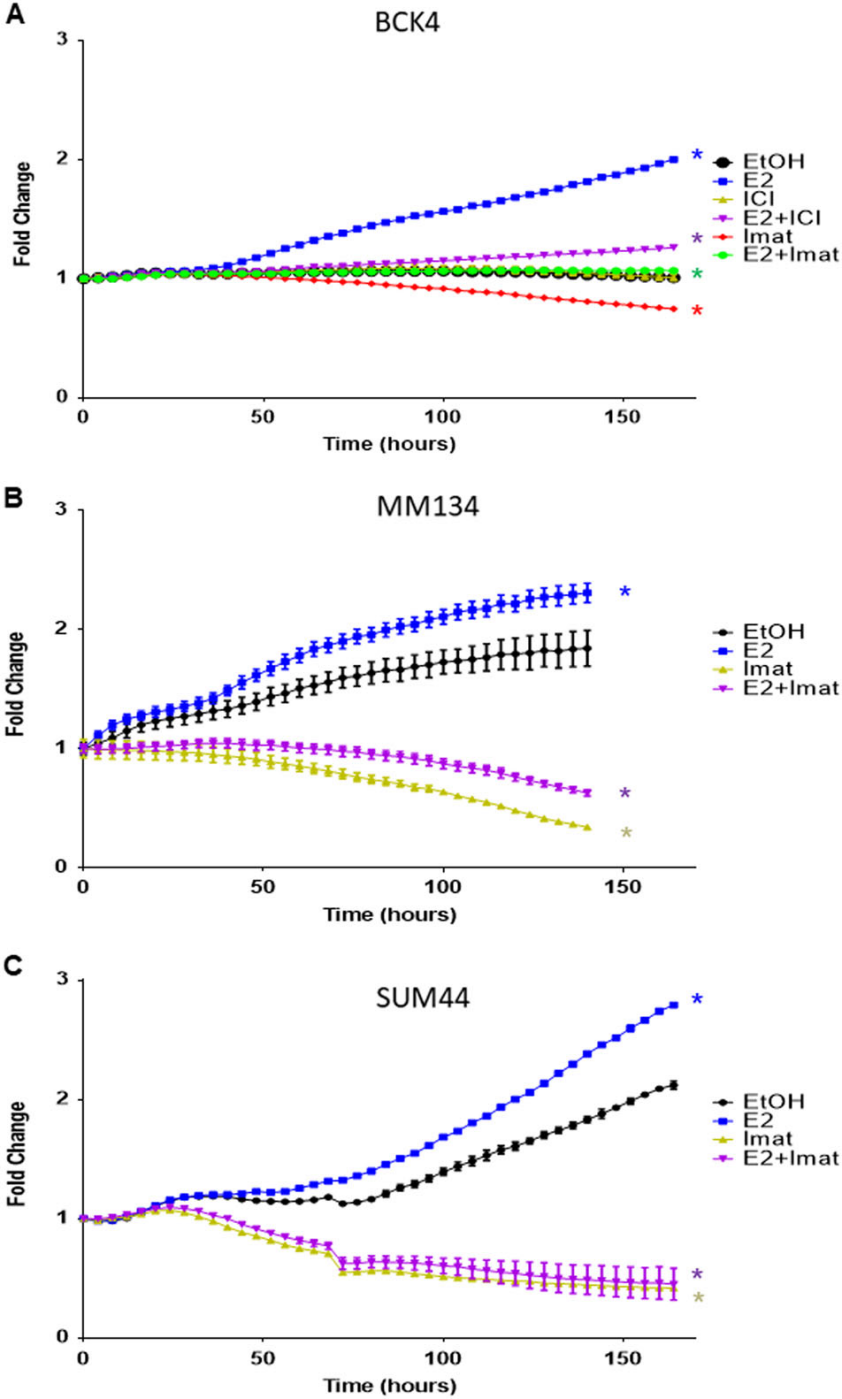
**a** BCK4 cells were placed in phenol red free media with steroid depleted serum 24 h prior to treatment with vehicle (EtOH), estradiol (E2), fulvestrant (ICI), imatinib mesylate (Imat) or combined treatments as specified. Proliferation was measured using the IncuCyte live cell imaging system. Fold change was calculated vs. time zero. **b** MDA-MB-134VI (MM134) or **c** SUM44PE (SUM44) cells were plated as specified above for 24 h prior to treatment with vehicle (EtOH), estradiol (E2), imatinib mesylate (Imat) or the combination of E2+Imat as specified. Proliferation was measured using the IncuCyte live cell imaging system for the times specified. Error bars show SEM. Asterisk indicates *p* < 0.0001 vs. vehicle control using two way ANOVA

**Fig. 5:**
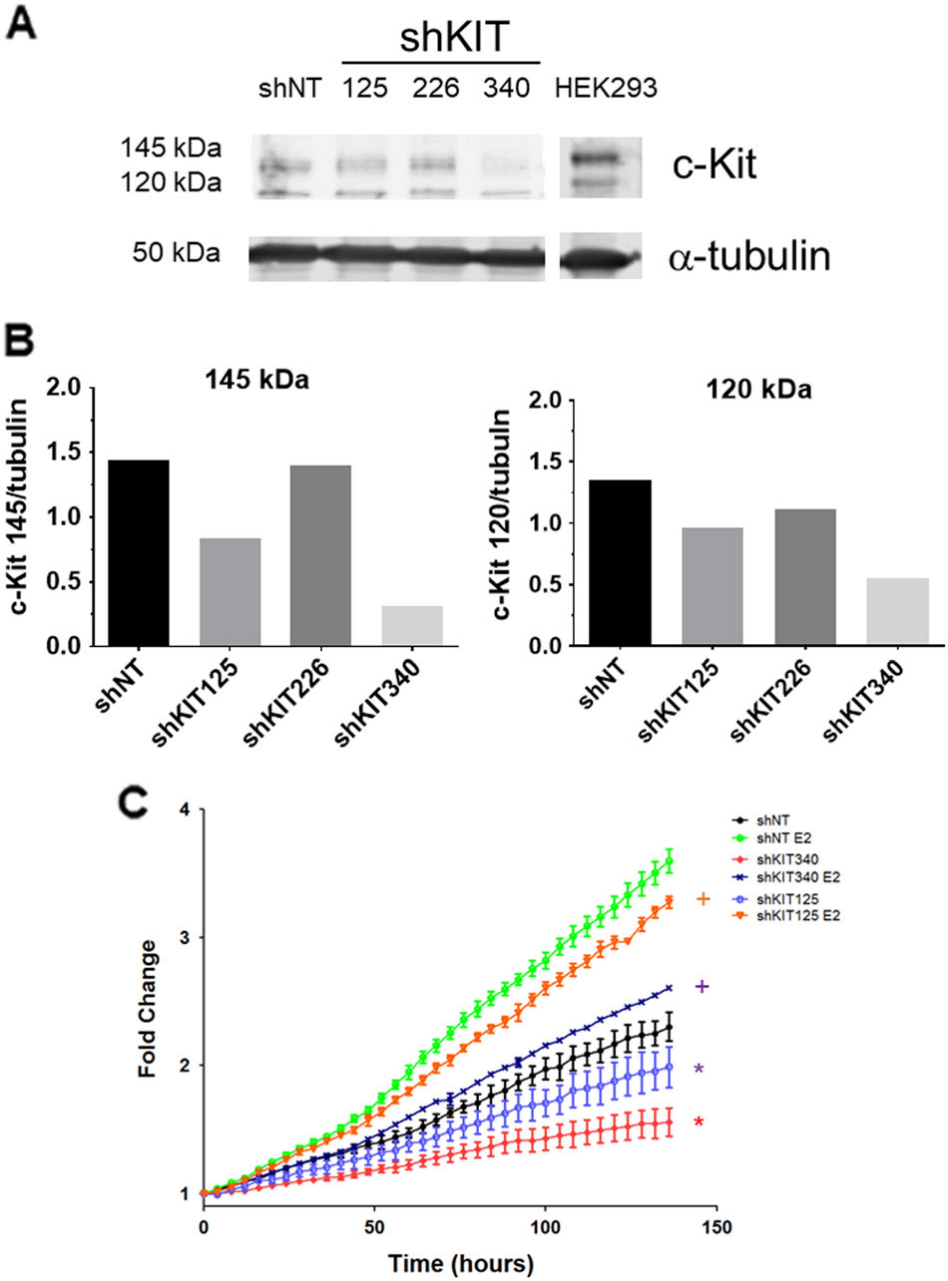
**a** Western blot showing diminished expression of c-Kit in BCK4 cells containing shRNA directed to c-Kit. Alpha tubulin is shown as a loading control. **b** Quantification of the 145 and 120 kDa isoforms of c-kit normalized to tubulin is shown. **c** Proliferation of BCK4 cells expressing a non targeting (shNT) or c-Kit targeted shRNAs treated with vehicle (EtOH) or estradiol (E2). Proliferation was measured using the IncuCyte live cell imaging system for the times specified. Asterisk indicates *p* < 0.0001 compared to shNT; “+” indicates *p* < 0.0001 compared to shNT E2 using two-way ANOVA

**Fig. 6:**
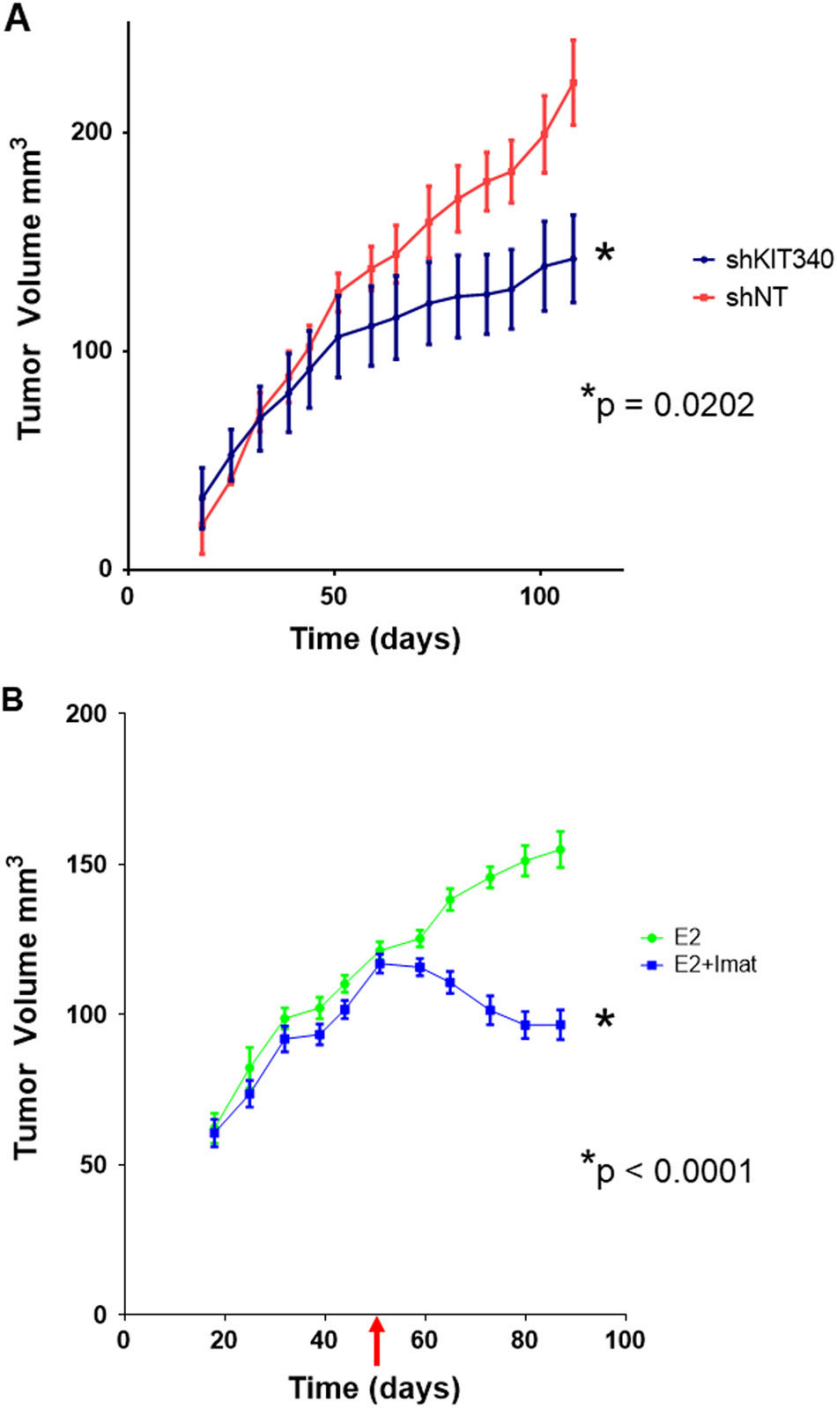
**a** BCK4 cells expressing a non-targeting control (shNT) or *c-Kit* targeted shRNA (shKIT340) were implanted into NSG mice supplemented with E2. Tumor volume was measured over 60 days. *N* = 5 tumors/group. Asterisk indicates *p* = 0.0202 and statistical significance was determined using a two tailed t-test. Error bars show SEM. **b** BCK4 cells were implanted into NSG mice supplemented with E2 and randomized to two groups, with or without imatinib mesylate (Imat). The red arrow shows commencement of treatment with imatinib on day 52. The tumor volume of mice treated with imatinib (blue line) or control (green line) is shown. *N* = 8 tumors/group. Asterisk indicates *p* < 0.0001 and statistical significance was determined using a two way ANOVA. Error bars show SEM

To confirm the importance of c-Kit on the biology of ILC, we used imatinib mesylate (Gleevec®), an inhibitor of c-Kit and several other receptor tyrosine kinases. Next we tested the ability of imatinib mesylate (imatinib) to inhibit proliferation of BCK cells. Figure 4a shows imatinib strongly inhibits proliferation of BCK cells, even in the presence of E2 when c-Kit expression is induced (Fig. 3b). We also examined the effects of imatinib on two other ER+ ILC cell lines, SUM44 and MM134, where it also strongly inhibits E2 stimulated proliferation (Fig. 4b and c). In addition to c-Kit, imatinib also inhibits the Discoidin Domain Receptor Tyrosine Kinase 1 (DDR1), platelet derived growth factor receptor (PDGFR) the ABL proto oncogene 1, and non receptor tyrosine kinase (c-Abl) with an IC50 of 97, 43, 100 and 600 nM, respectively^34–36^. To confirm the effects of imatinib on E2 induced proliferation are mediated by c-Kit, we created stable clones to decrease the levels of c-Kit using shRNA (Fig. 5a). The strongest decrease in c-Kit was mediated by shKIT340; second strongest was shKIT125 vs. the non-targeting (shNT) control (Fig. 5a and b). Next we treated the shKIT clones with or without E2 to observe effects on proliferation. BCK4 cells with the strongest decrease in c-Kit expression (shKIT340) showed a 33% and a 28% decrease in baseline and E2 induced proliferation respectively vs. the shNT control (Fig. 5c). Next we tested the effects of decreased c-Kit in vivo. We used BCK4 shNT or shKIT340 cells implanted into immunocompromised mice. BCK4 tumors with decreased expression of c-Kit show decreased proliferation vs. the non-targeting control by Ki67 labeling (Fig. 6a and Supplementary Fig. 6). We also tested the effects of imatinib treatment on BCK4 tumor growth. Once tumors reached 120 mm^3^ mice were randomized to receive either vehicle control or imatinib (100 mg/kg/day). Imatinib treatment slowed E2 dependent growth compared to vehicle-treated tumors (Fig. 6b). Taken together these data demonstrate that c-Kit regulates E2 dependent proliferation in BCK4 cells.

## Discussion

Some previous studies have shown dramatic differences among E2 regulated genes in ER+/PR+ breast cancer cell lines and their cognate xenograft tumors^37^, while other studies show ~ 40% overlap among genes regulated in cell lines and tumors^38^. Our studies suggest E2 regulated genes are similar in BCK4 cells and BCK4 derived tumors and we show many genes commonly regulated among ER+ ILC cell lines. This suggests the BCK4 cell line is a valid model for studying lobular breast cancers.

Connexin 43 (Cx43; GJA1) is a gap junction protein involved in cell-cell signaling via the transfer of small molecules. Cx43 is also upregulated by E2 in osteocytes^39^. In the murine uterus, E2 regulates Cx43 in stromal cells^40^ strongly during early pregnancy where Cx43 mediates stromal cell differentiation and tissue neovascularization^41^. In the normal mouse mammary gland Cx43 is expressed in myoepithelial cells and stroma^42^. Cx43 expression in breast cancer is regulated by mir206 where decreased Cx43 expression repressed proliferation and invasion of breast cancer cells^43^. Conversely, treatment of ER+ breast cancer cells with a small peptide that increased Cx43 activity decreased breast cancer cell proliferation and improved sensitivity to tamoxifen^44^, and Cx43 expression correlated with a good prognosis^45^. Mutating Cx43 in mice decreased total Cx43 levels and a developmental delay in puberty with dysfunction milk ejection^42^ and also increased mammary tumor metastases to the lung^46^, suggesting wild type Cx43 suppresses metastasis. However, Cx43 mediates transfer of cGAMP between breast cancer cells and astrocytes in brain metastases^47^ and Cx43 and Cx43 levels are higher in metastases than primary tumors^47^. Cx43 was upregulated by E2 both in BCK4 cells and tumors, and had the highest fold regulation among all genes in BCK4 tumors (51 fold, Supplementary Table 1) and Cx43 is also E2 regulated in SUM44 cells implicating it may play a role in ILC. Because Cx43 allows transfer of small molecules (glucose) and ions (Ca^2+^, K^+^) and small signaling mediators (IP3, cAMP) between cells, in the absence of e-cadherin, Cx43 may facilitate the informational flow between lobular breast cancer cells.

c-Kit is expressed in the normal mammary epithelium in both mice and humans where the majority of c-Kit+ cells lack ER. However, there is a subset of luminal progenitor cells that are both ER+ and c-Kit+ and c-Kit is required for proliferation of the normal mammary gland^48^. While c-Kit is highly expressed in normal breast epithelium, its expression during breast cancer is controversial. Studies examining c-Kit in breast tumors show c-Kit is rarely mutated and its expression ranges from 2.6% to 81%^15, 16, 19, 22, 49, 50^. Specifically in ILC, c-Kit expression is anywhere from 0.5–30% of all ILC^15, 19^ and 15.4% specifically in pILC^16^. C-Kit protein is also expressed in 53% of breast cancer cell lines^49^ and treatment with the ligand for c-Kit stimulated proliferation in MCF7, ZR75-1 and MDA-MB-231 cells^51^ and cells that overexpress c-Kit show increased growth and clonogenicity^52^. A proliferative role for c-Kit was confirmed in the BCK4 cells where a reduction in c-Kit decreases proliferation. This suggests targeting c-Kit in pILC may be a viable treatment for patients with c-Kit+ tumors. Our studies show ILC cells express two isoforms of c-Kit, (120 and 145 kDa). The specific function of each c-Kit isoform in breast cancer is unknown although the 145 kDa form appears to be the glycosylated version of the 120 kda protein^53, 54^. Importantly, the ligand for c-Kit, KITL, is also induced by E2 in BCK4 tumors. Thus it is likely there is an autoregulatory growth signaling in ILC tumors involving the c-Kit pathway that could be inhibited by targeting c-Kit by Imatinib (Fig. 6). Downstream pathway regulation mediated by c-Kit signaling is complex (reviewed in ref. ^55^); future studies will examine c-Kit signaling in these tumors.

The diffuse growth pattern of ILC within the breast makes resecting surgical margins difficult; consequently, many patients with ILC undergo mastectomy rather than breast conserving therapy. ILC also metastasize to unusual sites such as the ovary, peritoneum and gastrointestinal tract. These metastases can be clinically silent, or be mistakenly identified as other tumor types. Therefore treatments specific for metastatic ILC are needed clinically, especially because these tumors typically don’t respond to chemotherapy. The number of ILC metastases containing c-Kit is unclear. ILC are less responsive to specific types of endocrine therapy than IC-NST (reviewed in ref. ^27^) and are less responsive to chemotherapy (reviewed in ref. ^12^). Thus new therapies are needed for advanced ILC that are resistant to endocrine therapy and/or chemotherapy. Two small phase 2 clinical trials have assessed the efficacy of imatinib mesylate for treatment of advanced breast cancer^56, 57^, both showed no evidence for efficacy, perhaps because the majority of patients in these studies had PDGFR+ tumors and were negative for c-Kit+^56, 57^. A third ongoing trial examines the efficacy of combining imatinib with an aromatase inhibitor in patients with ER+ or PR+ and PDGFR+ or c-Kit+ metastatic breast cancer (NCT00338728). However, no clinical studies have examined efficacy of imatinib in treating patients with early breast tumors and/or patients with breast tumors that are ER+/c-Kit+. Our studies suggest patients with ER+/c-Kit+ ILC respond to treatment with imatinib.

## Materials and methods

### Cell lines

BCK4 and MCF7 cells were grown in MEM+5% FBS as previously described^13^. ZR75-1 cells were grown in RPMI+5% FBS. PT-12 cells were grown in DME/F12 + 10% FBS with cholera toxin and insulin. MDA-MB-134VI cells were purchased from the University of Colorado Tissue Culture Core and grown as described^27^. SUM44PE cells were purchased from Asterand and were grown according to distributor’s instructions. For hormone treatments, cells were placed in the following conditions: BCK4 and MCF7 cells in phenol red free MEM+5%DCC, NEAA and insulin (6 ng/mL), MDA-MB-134VI cells were placed in phenol red free DMEM/L15+10% DCC, and SUM44PE cells were placed in phenol red free F12+5%BSA for 48 h prior to treatment with vehicle (EtOH) or 10 nM 17-beta estradiol (E2) for times specified. For longer timepoints cells were treated every 72 h. All cell lines were mycoplasma negative (MycoAlert, Lonza) and authenticated by the University of Colorado Cancer Center Tissue Culture Core Laboratory using STR analysis.

### Stable cell lines

Lentiviral vectors encoding small hairpin RNA (shRNA) were used to stably inhibit c-Kit. A scrambled non-targeting vector was used as the negative control. Stably expressing cells were selected using puromycin. ShRNA vectors (Mission; Sigma) were from the University of Colorado Functional Genomics Facility (Aurora, CO, USA). Four constructs were screened (378437, 363125, 284340, 195226); two (284340 and 363125) were used for further analysis and abbreviated as shKIT340 and shKIT125, respectively.

### Immunoblotting

Whole cell extracts of cells were prepared and resolved as previously described^58^. Primary antibodies used include Cx43 (Abcam, ab11370), PR (Dako, PgR-1294), c-Kit (Santa Cruz Biotechnology, sc-168) or c-Kit (R&D systems, AF1356), ERα (SP1, Thermofisher, RM-9101) α-tubulin (Sigma-Aldrich, T5168), GAPDH (Cell Signaling, 2118S). Secondary antibodies included were Alexa-fluor 680 Goat-Anti-Mouse IgG (A21058; Lifetechnologies, Thermofisher, USA) and Alexa-fluor 680 Goat-Anti-Rabbit IgG (A21109; Lifetechnologies, Thermofisher, USA). Immunoblots were imaged using the Odyssey Infrared Imaging System (Li-Cor Biosciences). Experiments were repeated at least twice.

### Proliferation assays

Cell proliferation assays were performed using the IncuCyte live cell imaging system (Essen Biosciences) of either nuclear labeled (nuclear count) or unlabeled cells (percent confluency). Images were captured every 4 h; data (nuclear count or confluency) were exported and analyzed using PRISM software. Statistics were performed using student’s *t*-test. Experiments were repeated at least twice.

### Hormones and inhibitors

Imatinib mesylate was a gift of Novartis and was resuspended in sH20 and sterile filtered. Fulvestrant (ICI182,780) was purchased from Tocris Biosicence. 17beta estradiol (E2) was purchased from Sigma.

### Xenograft experiments

Experiments were performed under an approved IACUC protocol at the University of Colorado (83913(12)1E). BCK4 cells were grown as xenografts in 4–6 week old female NOD-SCID mice as previously reported^13^. For experiments using shNT or shKIT340, 4–6 week old female NOD-SCID gamma (NSG) mice were used and implanted with a pellet containing 2 mg of beta-estradiol. Tumor growth was measured weekly using digital calipers and tumor volume was calculated using (length x width^2^)/2. For imatinib treatment, mice were randomized into 2 groups based on tumor size as measured by calipers. Imatinib was added to the drinking water to afford 0.75 mg/ml for estimated dosing of 100 mg/kg/day. Using a Newton Sample Power Analysis, the sample size was calculated using the interference for means comparing two independent samples. Using the values of 1 for mu1 (control treated tumors), 0.75 for mu2 (imatinib mesylate or shRNA treated tumors respectively), a sigma value of 0.14 with a 2 sided test, an alpha value of 0.05, and a power of 0.8, the sample size was calculated as 5 mice per group. Animals were removed and excluded from the study when a body condition score of ≤2 was observed. For data collection/analysis investigators were blinded to treatments.

### Immunohistochemistry

Immunohistochemistry was performed on tumors as previously described^13^. Unmasking was performed using citrate buffer, pH 6.0. Antibodies used were Cx43 (Abcam, ab11370), ERα (SP1, Thermofisher, RM-9101), CK8/18 (Novocastra, NCL-5D3), Ki-67 (Novus Biologicals, NB500-170), and c-KIT (R&D Systems, AF1356). Images were captured using an Olympus BX40 microscope equipped with an Olympus DX73 camera and Cell Sens software. For Ki67 staining images were captured using the Aperio Digital Pathology system (Leica Biosystems), and samples were quantified using Imagescope software (Leica Biosystems). Mucicarmine and H&E staining were performed as previously described^13^.

LASER Capture Microdissection, microarrays on tumors: Five million BCK4 cells were injected into the 4th mammary gland of NOD-SCID mice under an approved IACUC protocol (83913(12)1E) and grown for 5 months. Tumors were resected, embedded in O.C.T (Tissue-Tek), frozen in N2, and sectioned on a cryostat. Two tumors were used to obtain duplicate samples. Three thousand cells from each tumor region were LASER captured using the ArcturusXT microdissection system, RNA was isolated using the PicoPure RNA isolation Kit (Arcturus) followed by cDNA preparation using the Ovation Pico WTA System and Encore Biotin Module kit as previously reported^59^. Affymetrix Human Genome U133 Plus 2.0 Arrays were used to measure gene expression.

### Gene expression microarrays

3×10^6^ BCK4 cells were plated into phenol red free MEM +5% DCC for 48 h prior to treatment with EtOH (vehicle) or E2 (10 nM) for 6 or 24 h. Cells were harvested and RNA prepared using the Qiagen RNA extraction kit. All microarray procedures including total cellular RNA isolation, amplification, and Cy3/Cy5 labeling of 1.5 μg of experimental and reference RNA were done as previously described^28^. Microarray hybridizations were performed using Agilent human oligonucleotide 4x44K custom designed gene chips.

### Microarray analyses

For the hierarchical cluster analysis that compared the BCK4 cells to a large cohort of established breast cancer cell lines (Fig. 1a), the data from 105 published gene expression arrays (GSE50470), along with the BCK4 arrays, which were performed on the same platform with identical methods, were downloaded. Probes that represent intrinsic genes that segregate the intrinsic subtypes^28^ were filtered to require the Lowess normalized intensity values in both sample and reference to be >10, and included probes with >70% good data, leaving 4409 probesets. Cluster version 3.0 was used to median center the data, and perform hierarchical clustering of genes and arrays. Java Treeview version 1.16r4 was used to visualize the data. Analysis of this dataset with the Differentiation Score predictor^30^ identified relative differentiation scores (−1 to +1) of all microarrays. Proliferation scores were obtained by averaging the values of 11 genes within the PAM50 algorithm that are known to be associated with proliferation. To contrast this data with a previous study that investigated invasive lobular cell lines^31^ (Fig. 1b), we downloaded the data from GSE50695 and then utilized Distance Weighted Discrimination^60^ to combine the two datasets, which were then median centered and hierarchical clustered with Cluster. Significance Analysis of Microarrays^61^ defined estrogen regulated genes that were present in the DWD combined dataset were used for this study. Raw data have been deposited in the GEO database (Accession number GSE101742).

### Statistical significance

Statistical significance was assessed using a 2-tailed Student *t* test or a two-way ANOVA with a Tukey’s multiple comparisons test using GraphPad Prism 6 software. *P* ≤ 0.05 was considered statistically significant.

## Supplementary material

Supplementary Figure Legends

Supplementary Figures

Supplementary Table 1

Supplementary Table 2

## References

[1] Haltas H (2012). Invasive lobular carcinoma with extracellular mucin as a distinct variant of lobular carcinoma: a case report. Diagn. Pathol..

[2] Rosa M, Mohammadi A, Masood S (2009). Lobular carcinoma of the breast with extracellular mucin: new variant of mucin-producing carcinomas?. Pathol. Int..

[3] Yu J, Bhargava R, Dabbs DJ (2010). Invasive lobular carcinoma with extracellular mucin production and HER-2 overexpression: a case report and further case studies. Diagn. Pathol..

[4] Weigelt B (2008). Refinement of breast cancer classification by molecular characterization of histological special types. J. Pathol..

[5] McCart Reed AE, Kutasovic JR, Lakhani SR, Simpson PT (2015). Invasive lobular carcinoma of the breast: morphology, biomarkers and 'omics. Breast Cancer Res..

[6] Iorfida M (2012). Invasive lobular breast cancer: subtypes and outcome. Breast Cancer Res. Treat..

[7] Frost AR (1995). The significance of signet ring cells in infiltrating lobular carcinoma of the breast. Arch. Pathol. Lab. Med..

[8] Steinbrecher JS, Silverberg SG (1976). Signet-ring cell carcinoma of the breast. The mucinous variant of infiltrating lobular carcinoma?. Cancer.

[9] Hull MT, Seo IS, Battersby JS, Csicsko JF (1980). Signet-ring cell carcinoma of the breast: a clinicopathologic study of 24 cases. Am. J. Clin. Pathol..

[10] Arpino G, Bardou VJ, Clark GM, Elledge RM (2004). Infiltrating lobular carcinoma of the breast: tumor characteristics and clinical outcome. Breast Cancer Res..

[11] Diab SG (1999). Tumor characteristics and clinical outcome of tubular and mucinous breast carcinomas. J. Clin. Oncol..

[12] Christgen M (2016). Lobular breast cancer: clinical, molecular and morphological characteristics. Pathol. Res. Pract..

[13] Jambal, P. et al. Estrogen switches pure mucinous breast cancer to invasive lobular carcinoma with mucinous features. * Breast. Cancer. Res. Treat.* **137**, 431–48 (2013). 10.1007/s10549-012-2377-x PMC3543987 23247610

[14] Eusebi V, Magalhaes F, Azzopardi JG (1992). Pleomorphic lobular carcinoma of the breast: an aggressive tumor showing apocrine differentiation. Hum. Pathol..

[15] Ciriello G (2015). Comprehensive molecular portraits of invasive lobular breast cancer. Cell.

[16] Monhollen L, Morrison C, Ademuyiwa FO, Chandrasekhar R, Khoury T (2012). Pleomorphic lobular carcinoma: a distinctive clinical and molecular breast cancer type. Histopathology..

[17] Lim E (2009). Aberrant luminal progenitors as the candidate target population for basal tumor development in BRCA1 mutation carriers. Nat. Med..

[18] Ulivi P (2004). c-kit and SCF expression in normal and tumor breast tissue. Breast. Cancer. Res. Treat..

[19] Simon R (2004). KIT (CD117)-positive breast cancers are infrequent and lack KIT gene mutations. Clin. Cancer. Res..

[20] Charpin C (2009). Quantitative immunohistochemical expression of c Kit in breast carcinomas is predictive of patients' outcome. Br. J. Cancer..

[21] Kashiwagi S (2013). c-Kit expression as a prognostic molecular marker in patients with basal-like breast cancer. Br. J. Surg..

[22] Bacchi LM, Corpa M, Santos PP, Bacchi CE, Carvalho FM (2010). Estrogen receptor-positive breast carcinomas in younger women are different from those of older women: a pathological and immunohistochemical study. Breast..

[23] Susruthan MRS, Jayanth V, Archana K, Viswanath P (2015). C-kit expression in breast carcinoma- A study of 62 cases of breast carcinoma. Interantional Journal of Recent Trends in Science and Technology.

[24] Eroglu A, Sari A (2007). Expression of c-kit proto-oncogene product in breast cancer tissues. Med. Oncol..

[25] Figueira MI, Cardoso HJ, Correia S, Maia CJ, Socorro S (2014). Hormonal regulation of c-KIT receptor and its ligand: implications for human infertility?. Prog. Histochem. Cytochem..

[26] Hah N, Kraus WL (2014). Hormone-regulated transcriptomes: lessons learned from estrogen signaling pathways in breast cancer cells. Mol. Cell. Endocrinol..

[27] Sikora MJ, Jankowitz RC, Dabbs DJ, Oesterreich S (2013). Invasive lobular carcinoma of the breast: patient response to systemic endocrine therapy and hormone response in model systems. Steroids..

[28] Prat A (2013). Characterization of cell lines derived from breast cancers and normal mammary tissues for the study of the intrinsic molecular subtypes. Breast. Cancer. Res. Treat..

[29] Harrell JC (2012). Genomic analysis identifies unique signatures predictive of brain, lung, and liver relapse. Breast. Cancer. Res. Treat..

[30] Prat A (2010). Phenotypic and molecular characterization of the claudin-low intrinsic subtype of breast cancer. Breast. Cancer. Res..

[31] Sikora MJ (2014). Invasive lobular carcinoma cell lines are characterized by unique estrogen-mediated gene expression patterns and altered tamoxifen response. Cancer. Res..

[32] Neve RM (2006). A collection of breast cancer cell lines for the study of functionally distinct cancer subtypes. Cancer. Cell..

[33] Reiner GC, Katzenellenbogen BS (1986). Characterization of estrogen and progesterone receptors and the dissociated regulation of growth and progesterone receptor stimulation by estrogen in MDA-MB-134 human breast cancer cells. Cancer. Res..

[34] Buchdunger E (1995). Selective inhibition of the platelet-derived growth factor signal transduction pathway by a protein-tyrosine kinase inhibitor of the 2-phenylaminopyrimidine class. Proc Natl Acad Sci USA.

[35] Heinrich MC (2000). Inhibition of c-kit receptor tyrosine kinase activity by STI 571, a selective tyrosine kinase inhibitor. Blood..

[36] Manley PW (2010). Structural resemblances and comparisons of the relative pharmacological properties of imatinib and nilotinib. Bioorg. Med. Chem..

[37] Harvell DM, Richer JK, Allred DC, Sartorius CA, Horwitz KB (2006). Estradiol regulates different genes in human breast tumor xenografts compared with the identical cells in culture. Endocrinology..

[38] Creighton CJ (2006). Genes regulated by estrogen in breast tumor cells in vitro are similarly regulated in vivo in tumor xenografts and human breast tumors. Genome. Biol..

[39] Ren J, Wang XH, Wang GC, Wu JH (2013). 17beta estradiol regulation of connexin 43-based gap junction and mechanosensitivity through classical estrogen receptor pathway in osteocyte-like MLO-Y4 cells. Bone.

[40] Grummer R, Chwalisz K, Mulholland J, Traub O, Winterhager E (1994). Regulation of connexin26 and connexin43 expression in rat endometrium by ovarian steroid hormones. Biol. Reprod..

[41] Laws MJ (2008). Gap junction communication between uterine stromal cells plays a critical role in pregnancy-associated neovascularization and embryo survival. Development.

[42] Plante I, Laird DW (2008). Decreased levels of connexin43 result in impaired development of the mammary gland in a mouse model of oculodentodigital dysplasia. Dev. Biol..

[43] Fu Y (2015). Hsa-miR-206 represses the proliferation and invasion of breast cancer cells by targeting Cx43. Eur. Rev. Med. Pharmacol. Sci..

[44] Grek CL (2015). Targeting connexin 43 with alpha-connexin carboxyl-terminal (ACT1) peptide enhances the activity of the targeted inhibitors, tamoxifen and lapatinib, in breast cancer: clinical implication for ACT1. BMC. Cancer..

[45] Teleki I (2014). Correlations of differentially expressed gap junction connexins Cx26, Cx30, Cx32, Cx43 and Cx46 with breast cancer progression and prognosis. PLoS ONE.

[46] Plante I, Stewart MK, Barr K, Allan AL, Laird DW (2011). Cx43 suppresses mammary tumor metastasis to the lung in a Cx43 mutant mouse model of human disease. Oncogene..

[47] Chen Q (2016). Carcinoma-astrocyte gap junctions promote brain metastasis by cGAMP transfer. Nature..

[48] Regan JL (2012). c-Kit is required for growth and survival of the cells of origin of Brca1-mutation-associated breast cancer. Oncogene..

[49] Hines SJ, Organ C, Kornstein MJ, Krystal GW (1995). Coexpression of the c-kit and stem cell factor genes in breast carcinomas. Cell. Growth. Differ..

[50] Yared MA, Middleton LP, Meric F, Cristofanilli M, Sahin AA (2004). Expression of c-kit proto-oncogene product in breast tissue. Breast. J..

[51] Roussidis AE, Theocharis AD, Tzanakakis GN, Karamanos NK (2007). The importance of c-Kit and PDGF receptors as potential targets for molecular therapy in breast cancer. Curr. Med. Chem..

[52] Hines SJ, Litz JS, Krystal GW (1999). Coexpression of c-kit and stem cell factor in breast cancer results in enhanced sensitivity to members of the EGF family of growth factors. Breast. Cancer. Res. Treat..

[53] Reith AD (1991). Signal transduction by normal isoforms and W mutant variants of the Kit receptor tyrosine kinase. EMBO. J..

[54] Yarden Y (1987). Human proto-oncogene c-kit: a new cell surface receptor tyrosine kinase for an unidentified ligand. EMBO. J..

[55] Lennartsson J, Ronnstrand L (2012). Stem cell factor receptor/c-Kit: from basic science to clinical implications. Physiol. Rev..

[56] Cristofanilli M (2008). Imatinib mesylate (Gleevec) in advanced breast cancer-expressing C-Kit or PDGFR-beta: clinical activity and biological correlations. Ann. Oncol..

[57] Modi S (2005). A phase II trial of imatinib mesylate monotherapy in patients with metastatic breast cancer. Breast. Cancer. Res. Treat..

[58] Badtke MM (2012). Unliganded progesterone receptors attenuate taxane-induced breast cancer cell death by modulating the spindle assembly checkpoint. Breast. Cancer. Res. Treat..

[59] Horwitz KB, Dye WW, Harrell JC, Kabos P, Sartorius CA (2008). Rare steroid receptor-negative basal-like tumorigenic cells in luminal subtype human breast cancer xenografts. Proc Natl Acad Sci USA.

[60] Marron JS, Todd MJ, Ahn J (2007). Distance-weighted discrimination. J. Am. Stat. Assoc..

[61] Tusher VG, Tibshirani R, Chu G (2001). Significance analysis of microarrays applied to the ionizing radiation response. Proc Natl Acad Sci USA.

