# Supplementary material for: Estrogen induces c-Kit and an aggressive phenotype in a model of invasive lobular breast cancer
Source: Oncogenesis. 2017 Nov 27;6(11):396. doi: 10.1038/s41389-017-0002-x (PMC5868054; doi:10.1038/s41389-017-0002-x)
Supplement: Supplementary file 1 — Supplementary Figure Legends [file 41389_2017_2_MOESM1_ESM.docx]

Supplementary Figures:

Supplementary Figure 1: BCK4 tumors in cellulose supplemented mice are pure mucinous while tumors from E2 supplemented mice are mixed mucinous. BCK4 tumors (Figure 2) were stained with mucicarmine and counterstained with apple green and imaged at 10x magnification.

Supplementary Figure 2: Venn diagram showing overlap among estrogen regulated genes in BCK4 cells and BCK4 tumor subregions.

Supplementary Figure 3: Enriched pathways in estrogen treated BCK4 tumors. MetaCore software was used to analyze genes differentially regulated in MUCp versus ILC.

Supplementary Figure 4: Western blot of BCK4 cells treated with or without Estradiol (E2) for 1, 2, 7 or 14 days as shown. Expression of c-Kit, ERα and Connexin 43 (Cx43) is shown. GAPDH is shown as a loading control. Densitometry was performed for 145 kDa and 120 kDa c-Kit normalized to GAPDH.

Supplementary Figure 5: BCK tumors from cellulose or E2 treated mice stained with c-Kit (DAB), Connexin 43 (Cx43, DAB) or co-stained with ERα (DAB) and CK8/18 (fast red). 20X magnification is shown.

Supplementary Figure 6: BCK4 cells expressing a non-targeting control (shNT) or *c-Kit* targeted shRNA (shKIT340) were implanted into NSG mice supplemented with E2 (Figure 6A). Tumors were resected and immunohistochemistry was performed with an antibody to Ki67; representative images are shown at 20x magnification (top), magnification bar = 100um. Ki67 staining of tumors was quantified using Aperio Image Analysis (bottom). Asterisk indicates p=0.0006 and statistical significance was determined using a two tailed t-test. N=5 tumors/group. Error bars show SEM.
