# Supplementary material for: Estrogen induces c-Kit and an aggressive phenotype in a model of invasive lobular breast cancer
Source: Oncogenesis. 2017 Nov 27;6(11):396. doi: 10.1038/s41389-017-0002-x (PMC5868054; doi:10.1038/s41389-017-0002-x)
Supplement: Supplementary file 2 — Supplementary Figures [file 41389_2017_2_MOESM2_ESM.pptx]

## Slide 1
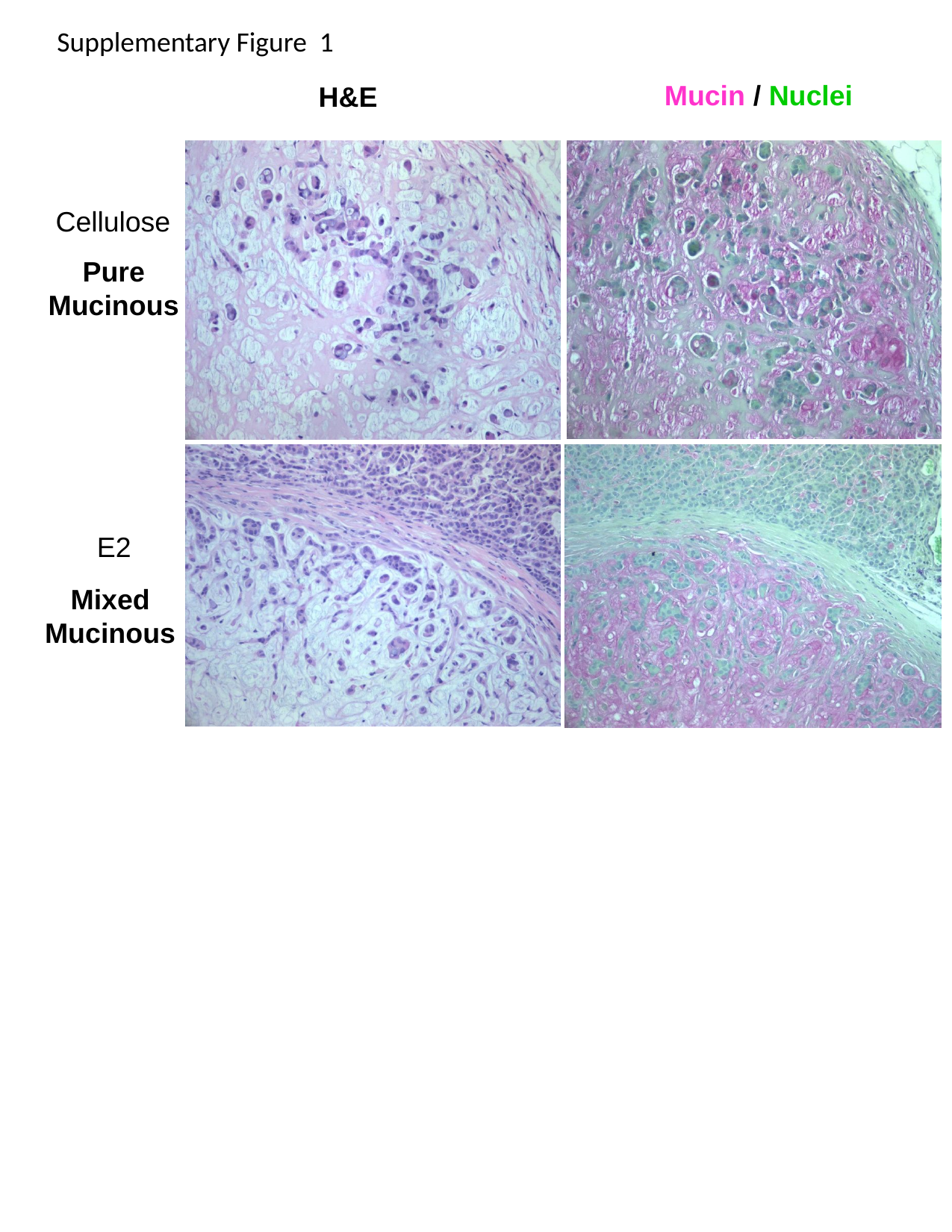

Supplementary Figure 1
Mucin / Nuclei
H&E
Cellulose
Pure
Mucinous
E2
Mixed
Mucinous

## Slide 2
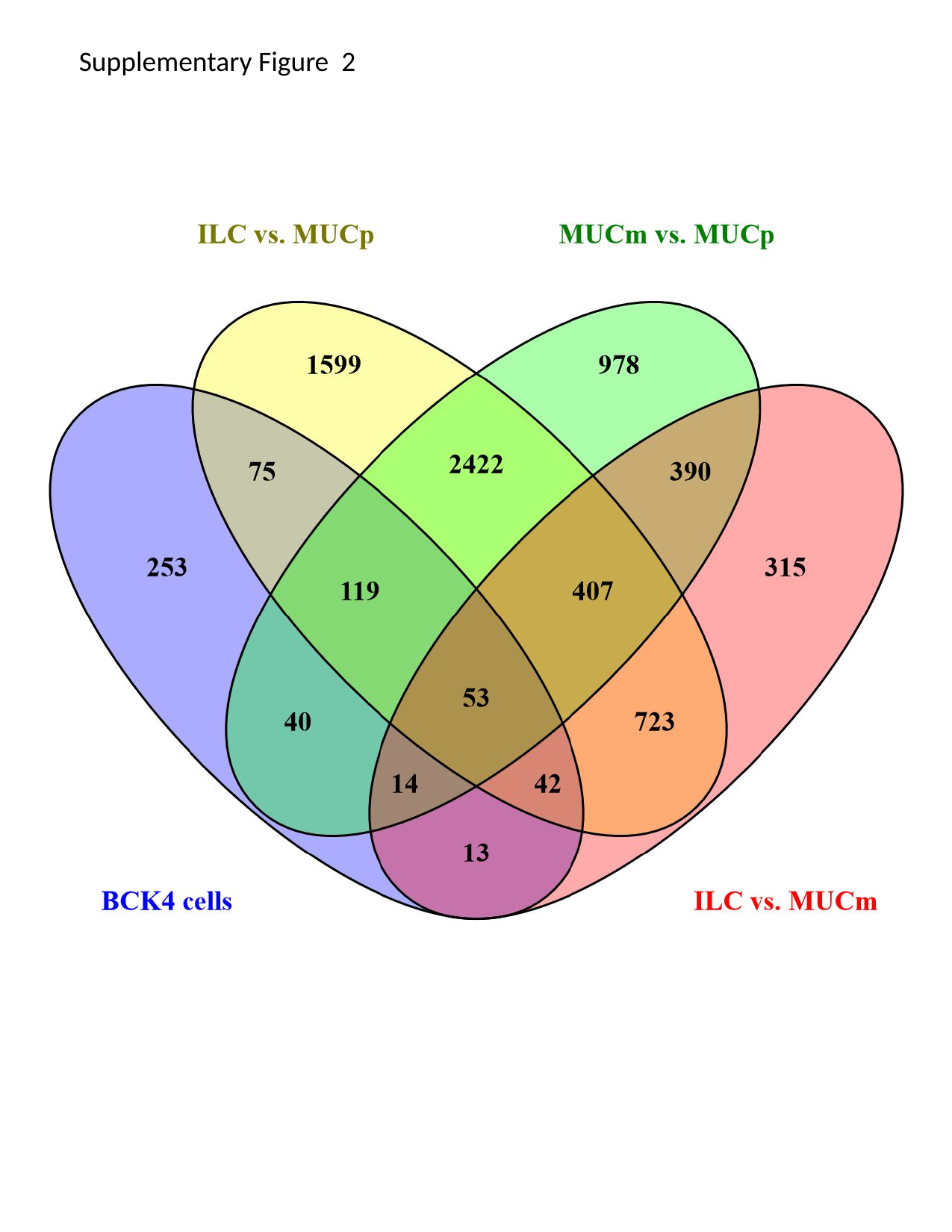

Supplementary Figure 2
MUCm vs MUCp
ILC vs MUCp
ILC vs MUCm
BCK4 Cells

## Slide 3
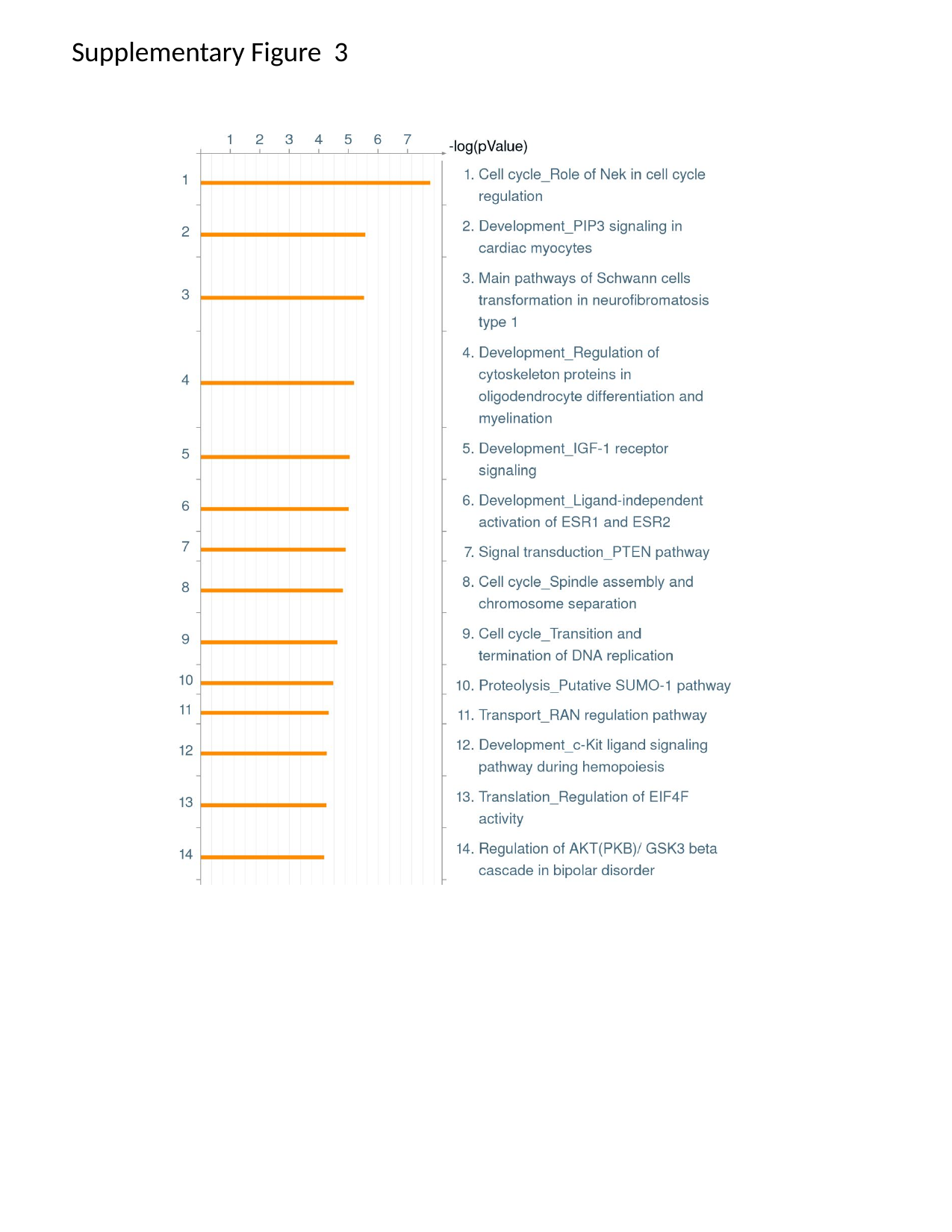

Supplementary Figure 3

## Slide 4
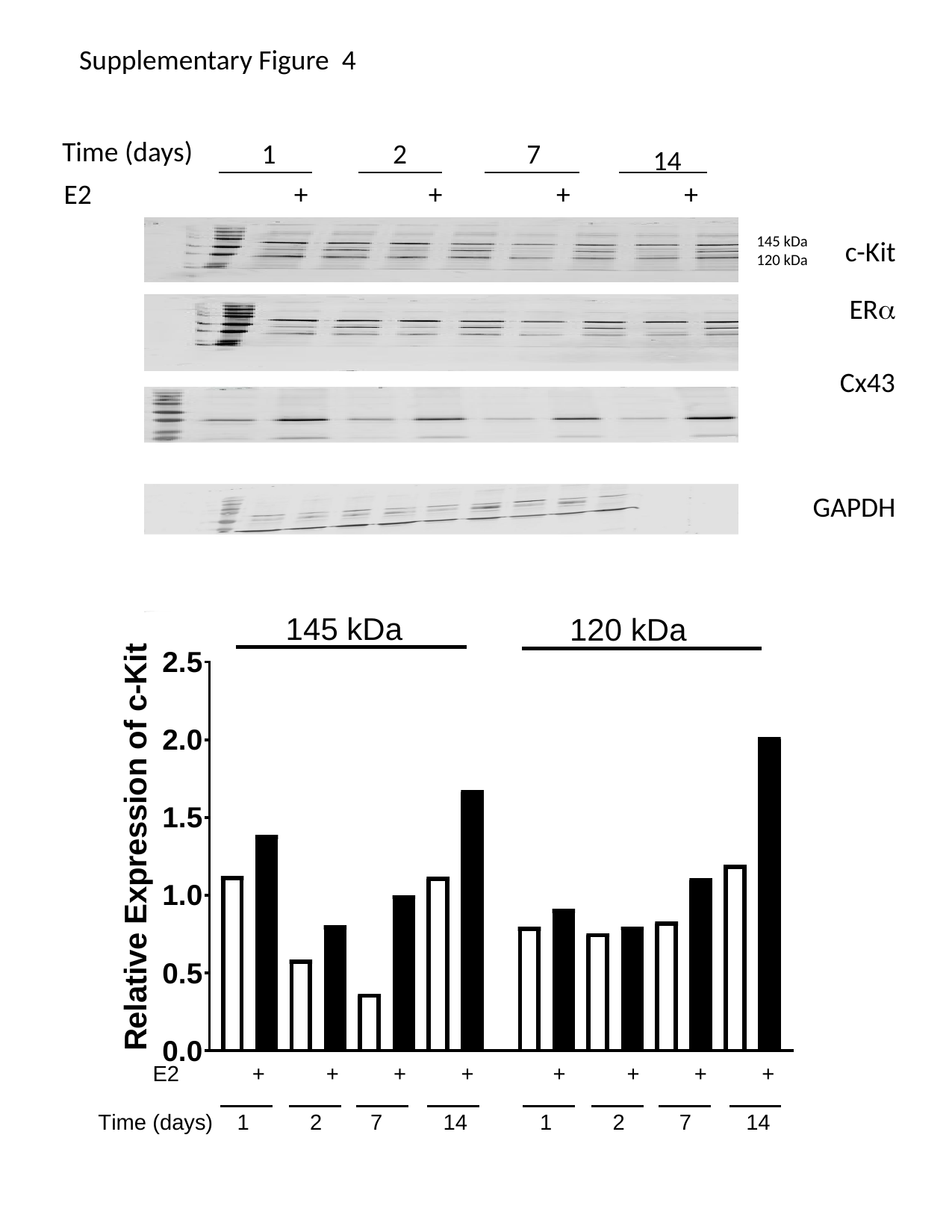

Supplementary Figure 4
Time (days)
1
2
7
14
E2 + + + +
145 kDa
120 kDa
c-Kit
ERa
Cx43
GAPDH

## Slide 5
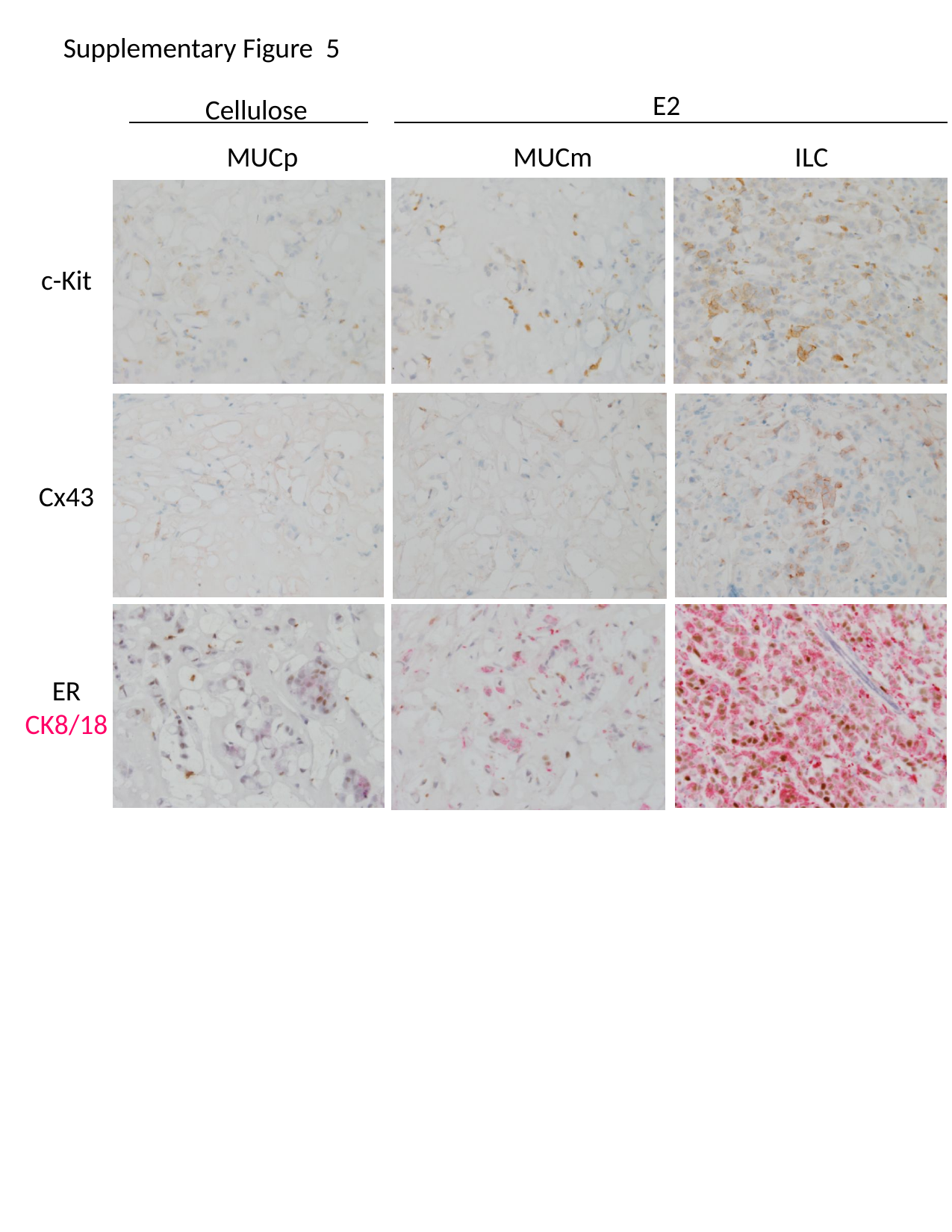

Supplementary Figure 5
E2
Cellulose
 MUCp MUCm ILC
c-Kit
Cx43
ER
CK8/18

## Slide 6
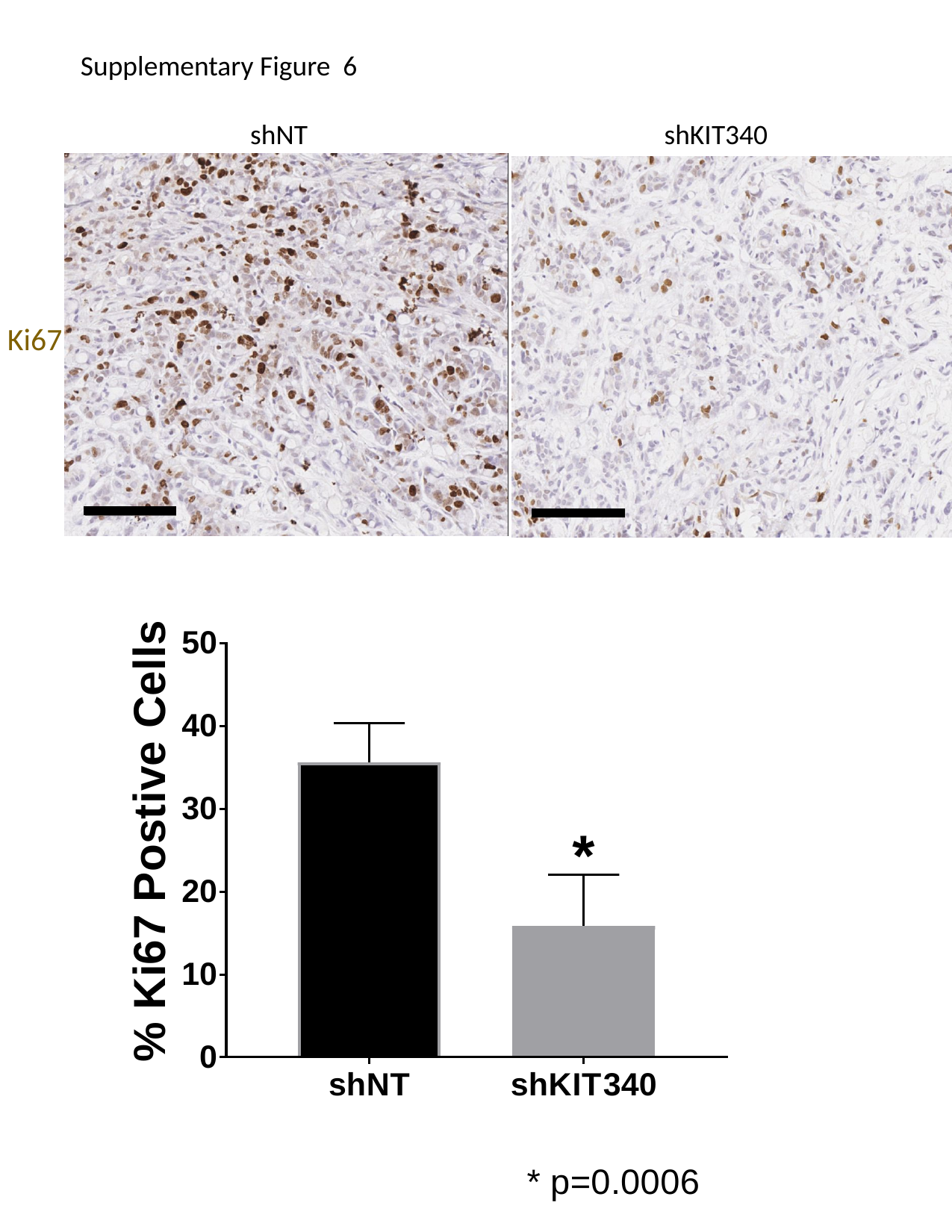

Supplementary Figure 6
shKIT340
shNT
Ki67
